# The potential action of SSRIs in the treatment of skin diseases including atopic dermatitis and slow-healing wounds

**DOI:** 10.1007/s43440-022-00423-7

**Published:** 2022-10-07

**Authors:** Aneta Kiecka, Marian Szczepanik

**Affiliations:** Chair of Biomedical Sciences, Faculty of Health Sciences, Institute of Physiotherapy, Jagiellonian University Medical College, Kopernika 7a, 31-034 Cracow, Poland

**Keywords:** Antidepressants, Atopic dermatitis, Selective serotonin reuptake inhibitors, Slow-healing wounds, Microbiota

## Abstract

Selective serotonin reuptake inhibitors (SSRIs) are used to treat affective and anxiety disorders. Antidepressants have also been shown to have antimicrobial and immunomodulatory effects, which may affect the microbiota-intestinal-brain axis. Studies show that SSRIs have antimicrobial activity both in vivo and in vitro and influence bacteria by inhibiting biofilm, affecting efflux pumps, among others. A huge challenge today is the prevention and treatment of skin diseases, including atopic dermatitis (AD) and slow-healing wounds. Skin diseases including AD and non-healing wounds are serious medical problem. People suffering from these conditions feel constant discomfort, which also affects their psychological state. Research on new treatments for AD and slow-healing wounds is essential because current medications are not fully effective and have many side effects. Exploring new drug groups for AD and slow-healing wounds will allow for the creation of an alternative treatment for these diseases. SSRIs represent a hope for the treatment of skin diseases due to their immunomodulatory and antimicrobial properties.

## Introduction

Selective serotonin reuptake inhibitors (SSRIs) are used to treat affective and anxiety disorders. Lewer et al. conducted a study in 27 European countries and found that SSRI consumption was about 7.2% in 2010 [1]. They have been shown to block serotonin (5-hydroxytryptamine, 5-HT) reuptake at the synaptic gap [2, 3]. Six SSRIs are commercially available: citalopram and its enantiomer escitalopram, fluoxetine, fluvoxamine, paroxetine and sertraline [4, 5]. Increasingly, antidepressants have also been shown to have antimicrobial and immunomodulatory effects which may affect the microbiota-intestinal-brain axis. The brain-intestinal axis is a bidirectional communication system between the central nervous system (CNS) and the gastrointestinal tract. Interestingly, SSRIs can affect the microbiota. The microbiota, on the other hand, can regulate CNS function mainly through the neuroimmune and neuroendocrine systems. It has been shown that bacteria in the intestines can produce various types of neurotransmitters that regulate host immune cell function via the nervous system. Studies suggest that host neurotransmitters and/or related pathways play a key role in the communication process. Additionally, O’Neill et al. found that the intestinal microbiome can influence skin homeostasis by regulating the coordinated differentiation of the epidermis and immune system function, although the mechanism is not yet fully elucidated [6–9].

A huge challenge today is the prevention and treatment of skin diseases, including atopic dermatitis (AD) and slow-healing wounds. These conditions are common, severe, and costly to treat [10]. It has been proven, among others, that non-healing wounds cause huge health care expenditures with a total estimated cost of over $3 billion per year in the United States [11]. Although an increase in morbidity is observed everywhere, rapidly developing countries are the most affected [12]. Effective therapies are still being sought to treat these conditions, as existing therapies are not fully effective and also may cause many side effects. The causes of these diseases are being investigated and the effect of certain factors on exacerbations is evaluated. SSRIs might be useful in the treatment of skin diseases due to their immunomodulatory and antimicrobial properties.

**Fig. 1:**
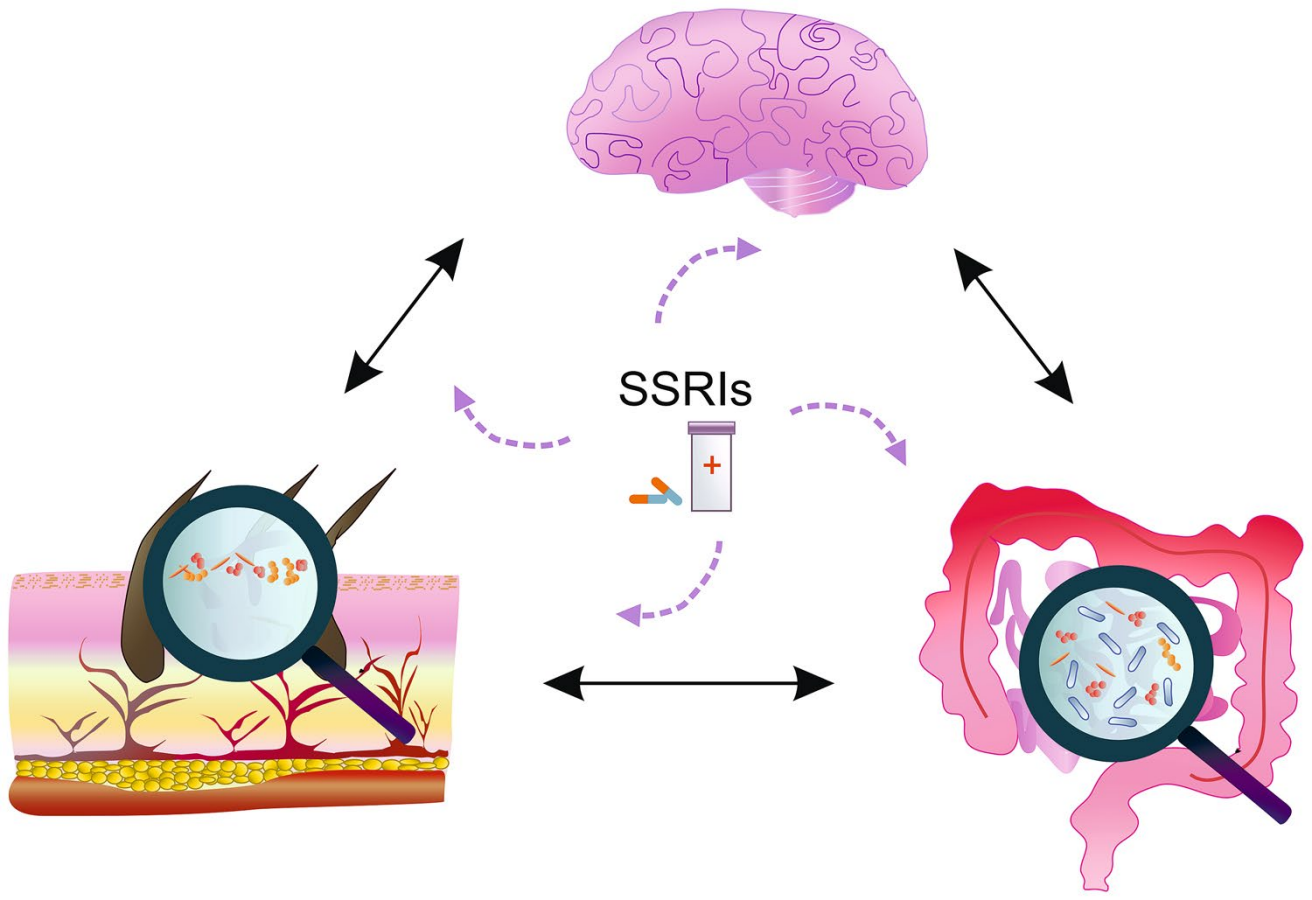
The brain-intestinal axis and SSRI. The brain-intestinal axis is a bidirectional communication system between the central nervous system (CNS) and the gastrointestinal tract. SSRIs can affect the microbiota. The microbiota can regulate the CNS function mainly through the neuroimmune and neuroendocrine systems. Bacteria in the intestines can produce various types of neurotransmitters that regulate host immune cell function via the nervous system. The intestinal microbiome can also influence skin homeostasis by regulating the coordinated differentiation of the epidermis and immune system function

This review collects data on the potential effects of SSRIs in the treatment of skin diseases including AD and slow-healing wounds (Fig. 1).

## Antimicrobial effect of SSRIs

In vivo and in vitro studies allow to determine the antimicrobial effect of drugs belonging to the SSRI group. It has been proven that sertraline and fluoxetine show the strongest antimicrobial activity [13]. The results show that fluoxetine administration has a significant effect on the diversity of intestinal microbiota in rats, e.g., there is a reduction in the genera *Prevotella*, *Oscillospira* and *Ruminococcus* [14]. Cussotto et al. found that rats treated with fluoxetine had an altered fecal microbiota composition and showed a decrease in *Deferribacteres* [15]. With respect to specific species, the minimum inhibitory concentration (MIC) values indicate that *Bacillus subtilis* and *Bacteroides fragilis* can be inhibited by sertraline or fluoxetine. In addition, it has been shown that SSRIs can also inhibit *Escherichia coli* and to a lesser extent inhibit *Clostridium* species, the bacteria present in the intestine [18]. In addition, sertraline significantly increases the antimicrobial effect of antibiotics, and some previously resistant strains become susceptible [16]. SSRIs including sertraline, fluoxetine and paroxetine are inhibitors of bacterial cell wall efflux and are effective against Gram-positive bacteria [16]. Furthermore, several studies have demonstrated the antifungal properties of sertraline, fluoxetine and paroxetine against *Aspergillus* spp*.* and *Candida* spp*.* [17, 18]. SSRIs were shown to have antimicrobial effects at high doses, while at low doses they enhance the antimicrobial effect, e.g., when used with antibiotics [19].

There are several possible mechanisms of antimicrobial effects of SSRIs. As mentioned, fluoxetine and sertraline may have stronger antimicrobial effects than other SSRIs. This may be related to their mechanism of action. Sertraline and fluoxetine are more hydrophobic than other SSRIs, thus they may be more easily passively diffused across the phospholipid membrane, allowing interaction with cellular machinery [20, 21]. In addition, SSRIs inhibit microbial efflux pumps that contribute to antimicrobial resistance [22]. The efflux pumps secrete antimicrobials and other drugs from the cytoplasm. Both sertraline and fluoxetine have been reported to inhibit efflux pump activity in *E. coli*, *Pseudomonas aeruginosa* and *Staphylococcus aureus* [23, 24]. Additionally, non-classical drugs such as SSRIs can exert an in vitro inhibitory effect and inhibit biofilm production in *Candida* spp*.* [25].

## Immunomodulatory effect of SSRIs

SSRIs also show immunomodulatory effects. Fluoxetine has been found to have anti-inflammatory effects in an experimental inflammation model [26]. Immune cells express various neurotransmitter receptors, thus antidepressants may regulate immune activity via interaction with these receptors. Interestingly, T lymphocytes playing a crucial role in adaptive immunity express 5-hydroxytryptamine (5-HT) receptors (5-HT1A and 5-HT2A/2C) [27, 28]. Many studies have investigated the effects of antidepressants in a treated depression on the immune response. In vitro studies have shown that various antidepressants such as tricyclics, SSRIs, and monoamine oxidase A inhibitors (moclobemide) can modulate the interferon-γ (INF-γ)/ interleukin-10 (IL-10) ratio [29]. Additionally, it has been shown that SSRI used to treat depression reduce levels of pro-inflammatory cytokines, including interleukin-1α (IL-1α), interleukin-6 (IL-6), interleukin-8 (IL-8), interleukin-12 (IL-12), and IFN-γ [30]. In a mouse model of lipopolysaccharide-induced sepsis, fluoxetine reduces mortality and decreases tumor necrosis factor-α (TNF-α) levels when used preventively. Fluoxetine has also been found to reduce lung inflammation in ovalbumin-sensitized rats, resulting in reduced numbers of macrophages, lymphocytes, eosinophils and neutrophils, and decreased expression of nuclear factor kappa B (NF-κB) [31]. Another study showed, decreased percentage of CD4^+^ T cells and increased percentage of CD8^+^ T lymphocytes after fluoxetine treatment, whereas the total percentage of CD3^+^ T cells was unchanged. The CD4^+^/CD8^+^ ratio was shown to be significantly decreased. Moreover, fluoxetine administration affected the interleukin-4 (IL-4)/interleukin-2 (IL-2) ratio, which was significantly increased in the fluoxetine-treated group compared with controls. Fluoxetine caused a significant decrease in cyclic adenosine monophosphate (cAMP) levels in lymphocytes probably through activation of serotonin receptors. Fluoxetine treatment modified immune parameters in plasma and rat lymphocytes [32]. Escitalopram and Paroxetine have been shown to reduce pro-inflammatory markers such as TNF-α, IL-1, IL-6, and PGE2 in addition to reducing depressive symptoms. Citalopram has also been shown to decrease the expression of CD4 coreceptors as well as chemokine receptors (CCR5, CXR4) in human immunodeficiency virus (HIV)-infected patients and thus inhibits viral entry into cells and replication [33].

## Immunomodulatory and antimicrobial effects SSRIs in atopic dermatitis

Atopic dermatitis is a chronic recurrent inflammatory skin condition. In industrialized countries, AD is diagnosed in approximately 15–20% of children and 1–3% of adults worldwide. AD has a major impact on patients’ quality of life, and the burden of direct and indirect costs (approximately $37.7 billion in personal costs worldwide) is borne by families and caregivers of AD patients [34]. Most AD cases are currently treated with topical anti-inflammatory agents such as topical corticosteroids and topical calcineurin inhibitors. Many patients can be successfully treated, but there is a group of people who continue to suffer from recurrent AD despite prescribed treatment. Studies show that drug therapies used for AD can cause various side effects including skin atrophy, telangiectasis, purpura, and stretch mark formation [35]. It has also been proven that AD patients often suffer from anxiety disorders and depression which can exacerbate the disease symptoms [36]. Therefore, there is a constant need to find a treatment with few side effects that is effective medication for AD. The hope is SSRIs, which in addition to their antidepressant effects, have immunomodulatory and antimicrobial effects that may be important in the treatment of AD.

### SSRIs inhibit the pruritus

One of the most common characteristic symptoms of AD is pruritus, defined as an unpleasant sensation that causes the need to scratch [37]. Long-term pruritus affects the quality of life of patients with AD. In many cases patients scratch the skin lesions causing erosions, ulcerations, bleeding that can aggravate the disease [38–40]. There are many causes of pruritus in AD including: genetic, environmental, and psychological factors [41]. The mechanism of pruritus is not fully understood. Although AD is associated with increased mast cell activation and histamine release, antihistamines are largely ineffective in treating AD-associated pruritus, indicating that pruritus in AD is nonhistaminergic [42]. SSRIs are sometimes used in the treatment of pruritus associated with AD. SSRIs including paroxetine and fluvoxamine have been shown to significantly reduce pruritus in AD patients [43–45]. Yaris et al. and Zylicz et al. suggest that the antipruritic effect of paroxetine may be mainly due to its central rather than peripheral action [46]. 5-HT is one of the key neurotransmitters that acts in both the central and peripheral nervous systems and stimulates skin fibroblast growth in a dose-dependent manner [47, 48]. Recent reports have shown that 5-HT induces melanogenesis through the 5-HT 2A (5-HT2A) receptor [49]. Both antipruritic and side effects of SSRIs correlate with increased serotonergic neurotransmission acting on postsynaptic receptors. Paroxetine may inhibit the 5-HT3 receptor, which may be related to the antipruritic effects of the drug. Chronic paroxetine therapy may modify central opioid receptors involved in itch signal processing, whereas acute effects may be related to an increase in serotonin acting on postsynaptic receptors [50]. In a study conducted by Fujimura et al. on canine atopic dermatitis, fluoxetine did not show efficacy in the treatment of atopic pruritus [51]. Another study showed that epicutaneous administration of fluoxetine in dogs did not reduce pruritus but had an effect on maintaining skin barrier integrity [52]. There are cases of individuals in whom increased serotonin levels exacerbate pruritus, but the responses in these patients may be due to high activity of the serotonergic system at the dermal and epidermal-dermal junction [53]. Paroxetine and fluvoxamine were tested in 72 patients with pruritus. Statistical analysis of the data showed the efficacy of paroxetine and fluvoxamine with no significant difference. The best response was observed in patients with pruritus caused by atopic dermatitis, systemic lymphoma, and solid carcinoma [45]. In addition, it has been shown that IL-31 may be involved in the mechanism of pruritus. IL-31 blood levels are elevated in many skin diseases with pruritus, including AD, cutaneous T-cell lymphoma, uremic pruritus, chronic urticaria and nodular pruritus. However, it is not known whether SSRIs affect the production of this cytokine which may be the subject of further research [42].

### *SSRI ameliorates atopic dermatitis *via* inhibition of IL-4 and IL-13 secretion and microbiota modification*

**Fig. 2:**
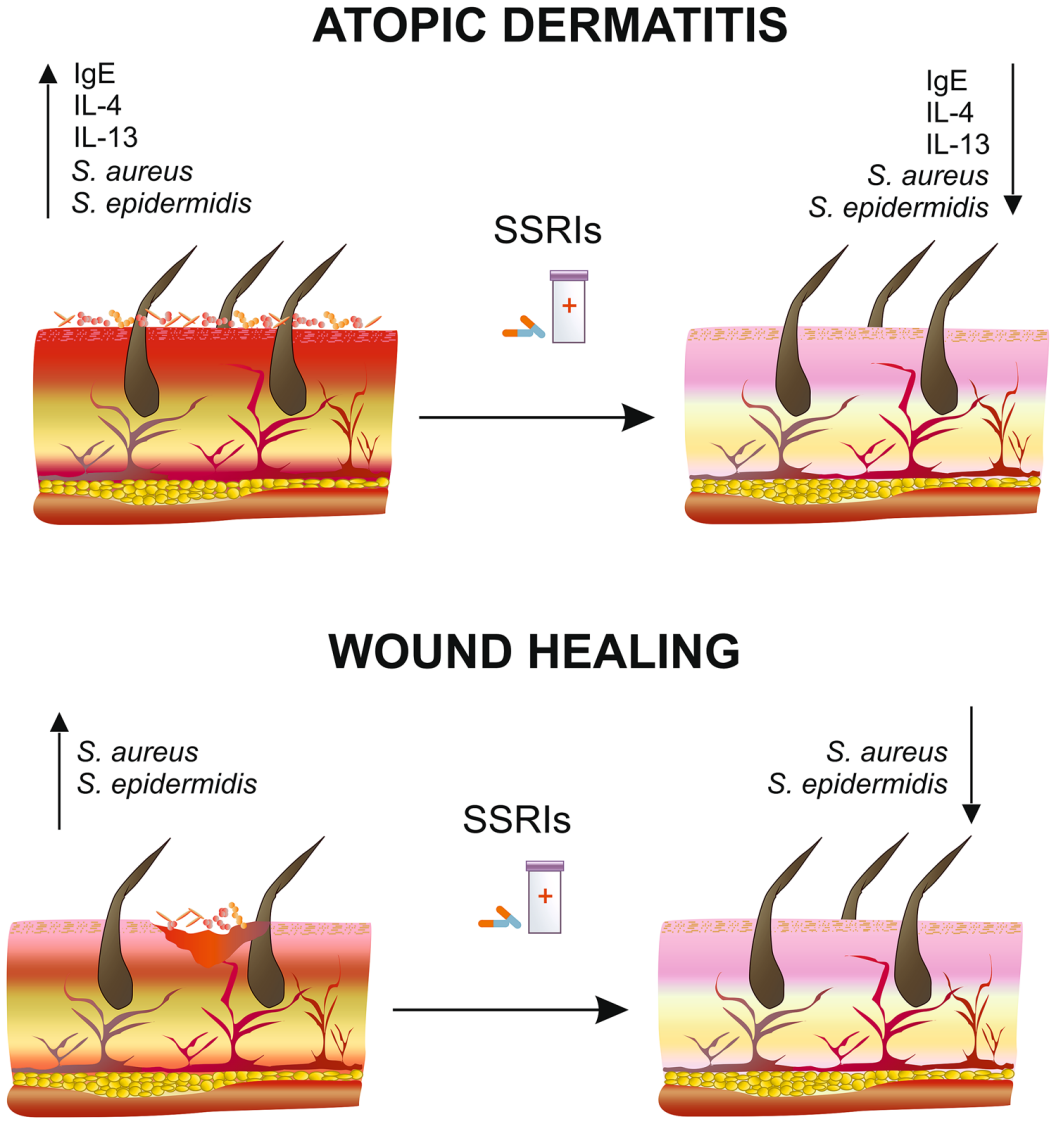
SSRI in atopic dermatitis and wound healing. It has been shown that atopic dermatitis (AD) patients have increased levels of serum immunoglobulin E (IgE) and cytokines such as IL-4 and IL-13 at the site of chronic dermatitis and skin colonization by *S. aureus* and *S. epidermidis*. Non-healing wounds are often colonized by *S. aureus* and *S. epidermidis*. SSRI treatment has been shown to modulate cytokine secretion (IL-4 and IL-13) and allergen-induced IgE production. SSRIs in vitro have activity against methicillin-resistant strains of *S. aureus* and *S. epidermidis*

It has been shown that AD patients have increased levels of serum immunoglobulin E (IgE) and Type 2 cytokines such as IL-4 and IL-13 at the site of chronic dermatitis [45, 54]. Serum IgE levels correlate with the severity of AD [55]. Studies show that treatment of mice with fluoxetine, venlafaxine and moclobemide inhibits humoral and cellular immunity with decreased release of pro-inflammatory mediators by macrophages and decreased expression of antigen presentation markers [56]. Interestingly, fluoxetine treatment restored normal serum IgE levels in AD patients. Furthermore, fluoxetine treatment has been shown to modulate cytokine secretion, including inhibition of allergen-induced IL-4 and IL-13 production (Fig. 2) [55]. In addition, fluoxetine significantly reduces lymphocyte proliferation as a result of increased central serotonin (5-HT) neurotransmission and activation of central 5-HT2 receptors [57]. Therefore, although the mechanism of amelioration of AD by fluoxetine remains unclear, the reduction of serum IgE and Th2 cytokine levels may be the main mechanism of action of fluoxetine [55]. It is also possible that anti-inflammatory and direct immunosuppressive effects of fluoxetine such as inhibition of T cell activation and proliferation, cytokine secretion, and induction of apoptosis observed in vitro and in vivo may be involved in AD alleviation [58, 59]. However, more studies are needed to fully understand the mechanism of action of fluoxetine and other SSRI that are involved in the amelioration of AD.

The intestinal microbiota constitutes a complex ecological system. It consists mainly of bacteria of the subtypes *Bacteroidetes* and *Firmicutes* (90%), and the types of *Actinobacteria*, *Proteobacteria* and *Verrucomicrobia*, which represent only its small part [60]. Other intestinal symbionts include fungi (especially yeasts) and some viruses [61]. In recent years, the composition of intestinal microbiota has been associated with many diseases. Numerous observations show that AD may be associated with the presence of a specific intestinal microbiome. Moreover, researchers have investigated whether there is a relationship between colonization by specific intestinal bacteria and the occurrence of AD. Studies show that low intestinal microbial diversity in the first month of life was related to the later onset of AD. After 12 months of life, when the microbiota had stabilized, Proteobacteria, containing Gram-negative organisms, were more common in infants without allergic symptoms [62]. In infants born by caesarean section, Enterobacteriaceae were found in significantly higher numbers in infants with eczema compared to infants without eczema. Bifidobacterium was detected in infants without eczema by 1.4 times more than in infants with eczema [63]. Storrø et al. tested whether microbiota profile in children affects the onset of AD. They showed that individuals who developed AD had lower levels of *B. fragilis* in youth [64], whereas Laursen et al. found no relationship between AD and intestinal microbiota during the first three years of life [65]. It has also been shown that higher Clostridium concentrations in the intestinal microbiota are associated with an increased risk of AD [66].

Studies show that SSRIs affect the intestinal microbiota leading to dysbiosis. The SSRI drugs such as sertraline, fluoxetine, citalopram and paroxetine have been shown to have antimicrobial effects in vitro on intestinal strains of *Staphylococcus*, *Enterococcus*, *Pseudomonas*, *Bacillus* and *Clostridium* [16]. The composition of intestinal microbiota in AD is not yet fully understood hence there are no studies that associate SSRI administration with modulation of intestinal microbiota in AD patients and thus possible inhibition of the disease. However, given the fact that SSRIs may affect intestinal *Clostridium* spp*.* the concentrations of which are higher in people with AD studies are needed to test this relationship. Additionally, with respect to specific species, MIC values indicate that *B. fragilis* may be inhibited by sertraline or fluoxetine. As described, this species is important in the intestine of individuals with AD. Furthermore, SSRIs have broad bactericidal effects and may modulate the intestinal microbiota of individuals with AD. With such modulation, oral administration of SSRIs may represent a possible new or complementary therapy for the treatment of AD.

Numerous studies have confirmed the association between skin colonization in AD subjects by *S. aureus* and the severity of AD symptoms [67]. A study using skin microbiota sequencing of children with AD showed that *S. epidermidis* may also be involved in AD pathogenesis. The AD relapses were also associated with a decrease in the diversity of skin microbiota [63]. Moreover, studies have shown that fluoxetine in vitro has activity against methicillin-resistant strains of *S. aureus* and *S. epidermidis* [68, 69]. Other studies show that AD is characterized by reduced barrier function, reduced activation of the innate immune response and susceptibility to *S. aureus* and reduced Gram-negative skin bacteria [70]. Interestingly, fluoxetine has been shown to enhance the effects of antibiotics against *P. aeruginosa* and *E. coli* and thus may be an adjunct to antibiotic therapy in wound infections of individuals with AD [71].

## SSRIs and wound healing

Slow-healing wounds are a major public health problem with high economic costs. Slow-healing or non-healing wounds are a significant cause of morbidity and mortality for a large proportion of the population. One of the major mechanisms responsible for the failure of chronic wound healing is an uncontrolled inflammatory response that is self-sustaining. Wound healing is a complex process involving the interaction of several cell types, cytokines, and other mediators. The wound healing process consists of four highly integrated and overlapping phases: hemostasis, inflammation, proliferation, and tissue remodeling or dissolution [72]. These phases and their biophysiological functions must occur in the correct order, at a specific time, and last for a specific period of time at optimal intensity [73]. Non-healing wounds affect approximately 3–6 million people in the United States, whereas the 65 years of age and older accounting for 85% of cases [11]. In addition, sometimes wounds become infected by pathogens which can further cause non-healing wounds. In recent years, it has been widely accepted that SSRI drugs can regulate inflammation and exhibit antimicrobial activity, and it has been suggested that they may play a role in wound healing [74].

### *SSRIs promote wound healing *via* immune response and microbiota modification*

T lymphocytes migrate into wounds following neutrophils and macrophages and reach a peak during the late proliferation/early remodeling phase. The role of T lymphocytes in wound healing is not fully understood and is currently an area of intense research. Several studies suggest that delayed T cell infiltration along with decreased T cell concentration at the wound site is related to impaired wound healing, while others report that CD4^+^ T cells (T helper cells) have a positive role in wound healing, whereas CD8^+^ T lymphocytes (Suppressor/cytotoxic T cells) play an inhibitory role in wound healing [75]. In the wound bed, fibroblasts produce collagen as well as glycosaminoglycans and proteoglycans, which are major components of the extracellular matrix (ECM). After vigorous cell proliferation and ECM synthesis, wound healing enters the final phase of remodeling, which can take years [76]. SSRIs may affect wound healing by interacting with various inflammatory mediators. Farahani et al. reported improved skin wound healing in chronically stressed rats by short- or long-term administration of fluoxetine [77]. Yuksel et al. suggested that short-term administration of paroxetine may improve skin wound healing by increasing the number of fibroblasts and causing better epithelialization over time in healthy rats [78]. Paroxetine has also been shown to enhance wound contraction to some extent which would potentially have either the property of contracting myofibroblasts or increase the number of myofibroblasts recruited to the wound area [79, 80]. These agents are also known to exhibit potent antiplatelet and endothelial protective effects. All data suggest that SSRI therapy can be considered as a potential wound care strategy [74]. Systemic administration of fluoxetine has been shown to exhibit anti-inflammatory properties in microglia, lymphocytes and other spleen cells. This topical therapeutic agent is believed to be a safe alternative to the serious clinical problem of chronic non-healing wounds [81].

So far, the therapeutic effects of SSRIs have been shown to be due to their anti-inflammatory and antimicrobial properties [82]. Howie et al. indicated that sertraline can slow bone healing while increasing the formation of mature collagen fibers [83]. Dwajani et al. found that paroxetine can promote wound healing [79, 80]. Numerous studies show that people with diabetes have an increased susceptibility to infections, this has been found in the diabetic wound healing model, among others [84]. One of the most common infections in people with diabetes is skin and soft tissue infections (SSTIs), which occur commonly as foot ulcers [85]. Patients with diabetes have impaired leukocyte function leading to insufficient migration of neutrophils and macrophages into the wound and reduced chemotaxis [86]. Redel et al. in their study using 16 s rRNA technique analyzed the skin microbiota composition of men with and without diabetes. They showed that although the microbiota composition and total bacterial counts were similar, a greater diversity of bacteria was observed in samples from men with diabetic healing wounds. Firmicutes count was lower in men with diabetes, whereas Actinobacteria count was higher. These wounds have also been shown to be frequently colonized by *S. aureus* [87, 88]. Chronic ulcers are associated with colonization by *S. aureus* and *P. aeruginosa* having surface proteins that affect wound healing [89]. In a mouse model of wound healing, the extracellular adhesion protein (Eap) of *S. aureus* has been shown to inhibit wound healing by interfering with host defense and repair mechanisms [90]. Higher glucose levels in diabetic individuals were found to increase *S. aureus* virulence, which was confirmed by the acquisition of two additional glucose transporters: GlcA and GlcC [91]. Additionally, also in individuals without diabetes, the bacteria colonizing chronic wounds are mainly *Staphylococcaceae* and *Pseudomonadaceae*, regardless of the etiology of the wound [92]. In a retrospective study, an analysis of wound microbiota of 2963 patients showed that the predominant species colonizing chronic wounds were mainly *S. epidermidis* and *S. aureus* and species such as *Corynebacterium* and *Propionibacterium* (Fig. 2) [93]. Interestingly, studies reveal that fluoxetine has an in vitro effect against methicillin-resistant strains of *S. aureus*. Fluoxetine causes bacterial death after plasma membrane damage. This fact may show new pathways by which SSRIs affect wound healing—through antimicrobial activity [69]. Fluoxetine and paroxetine have shown antimicrobial activity against standard strains of *S. aureus* ATCC 25,923, *E. faecalis* ATCC 51,299, and *S. epidermidis* ATCC 12,228 and clinical isolates of *S. aureus* in studies, thus they may be important in the healing of slow-healing wounds. It is potentially necessary to study the effect of epidermal administration of SSRIs in individuals with non-healing wounds to determine their effect on eliminating pathogenic bacteria and thereby accelerating the wound healing process.

## Conclusions

SSRIs constitute a group of drugs used for depression and anxiety disorders. However, there are new studies emerging to identify new properties of these drugs, i.e., their potential immunomodulatory and antimicrobial capabilities. Studies show that SSRIs affect bacteria by inhibiting biofilm, affecting efflux pumps, among others. Studies reveal that they have antimicrobial activity both in vivo and in vitro. They can modulate, e.g., the intestinal microbiota by affecting, among others, bacteria of the genus *Clostridium* spp*.*, a component of the intestinal microbiota. An additional reason that new drugs are being tested for potential antimicrobial activity is the ever-increasing resistance of pathogens to antibiotics. Studies show that the use of both an antibiotic and an SSRI enhances antimicrobial activity.

Skin diseases including AD and non-healing wounds are serious medical problem. People suffering from these conditions feel constant discomfort, which also affects their psychological state. Potentially, SSRIs may have an indirect influence through immunomodulation and modified microbiota on AD and wound healing. On the one hand, it may affect immunomodulation and microbiota composition; on the other hand, it may improve the mood of people with AD and healing wounds, which also may be partly related to its immunomodulatory properties. This gives the impression of a relationship between the brain-intestinal-microbiota axis involving the immune system. Further studies are needed to determine the antimicrobial properties of SSRIs in wound care. In addition, the exact mechanism by which SSRIs may inhibit pruritus in AD needs to be determined, among others, whether SSRIs affect the production of IL-31, a cytokine that seems to be important in the regulation of pruritus sensation in AD.

Research on new treatments for AD and slow-healing wounds is essential because current medications have many side effects and limitations. Exploring new drug groups for AD and slow-healing wounds will allow for the creation of an alternative treatment strategy for these diseases. However, the emergence of new therapies still requires the study of their exact mechanism of action.

## Data Availability

Not applicable.

## Abbreviations

AD: Atopic dermatitis
cAMP: Cyclic adenosine monophosphate
ECM: Extracellular matrix
IgE: Immunoglobulin E
INF-γ: Interferon-γ
IL-2: Interleukin-2
IL-4: Interleukin-4
IL-6: Interleukin-6
IL-8: Interleukin-8
IL-10: Interleukin-10
IL-12: Interleukin-12
MIC: Minimum inhibitory concentration
NF-κ: Nuclear factor kappa B
SSRIs: Selective serotonin reuptake inhibitors
